# Efficacy of indirect ELISA for serodiagnosis of melioidosis using immunodominant antigens from non-pathogenic *Burkholderia thailandensis*

**DOI:** 10.1186/s40064-016-3505-6

**Published:** 2016-10-19

**Authors:** Sumet Wajanarogana, Kanyanan Kritsiriwuthinan

**Affiliations:** 1 Department of Basic Medical Science, Faculty of Medicine, Vajira Hospital, Navamindradhiraj University, Bangkok, 10300 Thailand; 2 Faculty of Medical Technology, Rangsit University, Muang Ake, Pathumthani, 12000 Thailand

**Keywords:** Melioidosis, Flagellin, Outer membrane protein A, *B. thailandensis*

## Abstract

Melioidosis caused by gram negative bacteria, *B. pseudomallei*, is a fatal disease in the tropical and sub-tropical regions. However, sporadic cases have been reported in elsewhere. Early detection is imperative to reduce the mortality rate. Serological tests have being substantially developed using recombinant proteins as specific targeted antigens to melioidosis antibodies. In the present study, we focus on a truncated flagellin fragment (FLAG300) and outer membrane protein A (OmpABT) of *B. thailandensis* E264 as potential antigens for developing indirect ELISA to improve the serodiagnosis of melioidosis. Recombinant proteins were overexpressed and purified by immobilized metal affinity chromatography with denaturing conditions. The sensitivity and specificity of individual test were calculated within culture-confirmed melioidosis sera (n = 42) and non-melioidosis serum samples (n = 241) using the cut-off point at average of absorbance plus 2 standard deviations. The results demonstrated that a FLAG 300 based indirect ELISA showed 90.48 % sensitivity and 87.14 % specificity and an OmpABT based this assay revealed sensitivity of 80.95 % and specificity of 89.21 %. Their use in a double-antigen ELISA resulted in improve specificity (92.95 %) and still high degree of sensitivity (85.71 %). These data suggest a facile method for serodiagnosis of melioidosis by the use of antigens from a non-pathogenic strain.

## Background

*Burkholderia pseudomallei* infection, melioidosis, is an infectious disease that is mainly described in the major endemic areas of SE Asia and Northern Australia. However, there have been reported cases outside these endemic regions such as in Brasil, India and United States of America (Corral et al. 2008; Deshmukh and Mundhada 2013; Kunnathuparambil et al. 2013; Miralles et al. 2004). It is a major public health problem in Southeast Asia and Northern Australia (Dance 1991; Leelarasamee and Bovornkitti 1989), because the mortality rate is still high even with treatment, and no effective vaccine is available so far. The clinical manifestations of this disease are diverse, ranging from chronic localized infection in many organs to acute septicemia, complicating the clinical diagnosis of melioidosis. In septicemic cases, expiration will occur in a few days; thus reliable and early diagnosis is imperative. Although bacterial isolation and identification is the definitive diagnostic method, it is a time consuming strategy (5–7 days) (Cheng 2010). Moreover, bacterial culture is an imperfect reference method because it has low sensitivity and negative predictive value (Limmathurotsakul et al. 2010). Serological diagnosis of melioidosis is widely used in endemic regions, especially the indirect hemagglutination assay (IHA); however, it has limited utility for diagnosis because of relatively poor sensitivity (Appassakij et al. 1990; Harris et al. 2009; Sorenson et al. 2013) and a high seropositivity background in endemic areas (Ashdown and Guard 1984; Kanaphun et al. 1993). Although it is of limited value for diagnosis in those areas, it may be of assistance in some circumstances, particularly when paired sera are available or high serum titers are reported with the presence of clinical signs. Currently, a number of serodiagnostic tests for melioidosis detection has been developed using crude extracts (Sorenson et al. 2013; Cooper et al. 2013; Parthasarathy et al. 2006) or purified recombinant proteins from *B. pseudomallei* (Allwood et al. 2008; Arora et al. 2015; Chantratita et al. 2007; Chen et al. 2003; Druar et al. 2008) as antigens. Even though they obviously demonstrate substantial improvement over the clinical standard IHA test, all antigens are derived from a Tier 1 agent, *B. pseudomallei*, and thus manipulations must be done under biosafety level 3 (BSL-3). Conversely, such experiments with a close relative of this bacterium, *B. thailandensis*, can dispense with this biosafety level (Rotz et al. 2002). Both of the organisms are broadly similar genetically and immunologically (Yu et al. 2006). Previous studies indicate that melioidosis protective antibodies can be raised using antigens from *B. thailandensis* (Ngugi et al. 2010; Scott et al. 2013) and certain antigens of *B. pseudomallei* share epitopes with this closely related species (Wajanarogana et al. 2013). In addition, some biological properties of *B. pseudomallei* have been explained by using *B. thailandensis* as a non-pathogenic model (Haraga et al. 2008).

A number of serodiagnostic antigens of *B. pseudomallei* have been revealed using protein microarray (Felgner et al. 2009) and indirect enzyme-linked immunosorbent assays (ELISA) (Allwood et al. 2008; Arora et al. 2015; Wajanarogana et al. 2013; Hara et al. 2013). The majority of such antigens are cell surface-associated proteins such as outer membrane proteins (Omps), and secreted molecules. In this study, we focused on Omps, especially outer membrane protein A (OmpA) and truncated flagellin fragment (FLAG300) of *B. thailandensis* E264. The FLAG300 indicates as the potential antigen for diagnosis of melioidosis (Wajanarogana et al. 2013); however, culture-confirmed melioidosis serum samples have not yet been studied. As well as a flagellin fragment, OmpA of *B. pseudomallei* has immunogenic properties which has been used for serodiagnosis of melioidosis (Allwood et al. 2008; Arora et al. 2015; Hara et al. 2013). Moreover, both of these antigens have been suggested as potential candidate vaccines (Brett and Woods 1996; Hara et al. 2009; Ye and Gan 2007). Our preliminary study has found that *ompA* sequences of *B. pseudomallei* and closely related species show high homologues. Here, we isolate *ompA* from *B. thailandensis* E264 and express and purify it as recombinant protein for developing serodiagnosis of melioidosis, together with FLAG300.

## Methods

### Bacterial strains, cloning vector and culture condition

The *OmpA* gene and the truncated of flagellin gene of *B. thailandensis* E264 were inserted into expression vector, pET-24a(+) (Novagen). *E. coli* BL21(DE3) and XL1-Blue harboring recombinant expression vectors were basically grown in Luria–Bertani (LB) medium containing kanamycin (50 µg ml^−1^) at 37 °C with shaking.

### Serum specimens

Sera were used in this study from the following three groups of individuals: (1) Culture confirmed-positive of melioidosis serum samples collected from endemic regions, Amnat Charoen hospital and Khon Khaen hospital, Thailand (n = 42). (2) Serum samples from septicemic patients caused by other infectious bacteria (i.e. *Klebsiella pneumoniae*, *Escherichia coli*, *Pseudomonas aeruginosa*, gram positive bacteria and gram negative non-fermenter bacteria) (n = 74) collected from Thammasat hospital, Thailand. This group was used as the disease control. (3) Normal sera collected from healthy blood donor served as the negative control group (n = 167). The last group was obtained from Thammasat hospital (n = 49) and Amnat Charoen hospital (n = 118), Thailand. All sera in this study are leftover specimens of routine laboratory without direct contact with the patients and personal data record. Collection of serum samples was approved by the Rangsit University Ethics Committee (No. RSEC 17/35).

### OmpA gene isolation and cloning

The nucleotides sequence of *omp*A (BPSL2522; Sanger Research Institute, http://www.sanger.ac.uk) was used to design oligonucleotide primers to amplify *omp*A from *B. thailandensis* E264 with incorporated restriction sites. Forward primer, OmpAF (5′-CGCATATGAATAAACTTTCAAAGCTC-3′) and reverse primer, OmpAR (5′-GAATTCGCCTTCGCCG GAACG-3′), that introduced *Nde*I and *Eco*RI endonuclease sites (underlined), respectively, were custom synthesized from Bio Basic Inc., Canada. The polymerase chain reaction (PCR) was carried out with 100–200 ng of DNA template, 20 pmol of each primer, 1× *Pfu* buffer with MgSO_4_, 0.5 µl of 20 mM dNTPs, and 1.25 U of *Pfu* DNA polymerase (Fermentas, Life Sciences). Prior to DNA amplification, the mixture was heated at 99 °C for 5 min. The conditions for PCR amplification were as follows: one cycle of denaturation at 95 °C for 5 min, annealing at 60 °C for 1 min and extension at 72 °C for 1 min, 34 cycles of denaturation at 95 °C for 1 min, annealing and extension as the same previous condition, and the final cycle at 72 °C for 5 min. The PCR product was digested with *Nde*I plus *Eco*RI (Biolab) and cloned as an *Nde*I-*Eco*RI fragment downstream of the T_7_ promoter in the expression vector, pET24a(+), designated pET24a-OmpABT. The BL21(DE3) strain was transformed with pET24a-OmpABT recombinant plasmids.

### DNA sequencing and analysis

The nucleotides sequence of the selected clone was custom carried out in both orientations by Ward Medic Ltd. Part, Thailand. The sequence was analyzed by searching for homology in the entire database by nucleotide type of similarity search (blastn) with the BLAST program (Altschul et al. 1997) at the NCBI server (http://www.ncbi.nlm.nih.gov/BLAST/) and compared with the OmpA sequence (BPSL2522) of *B. pseudomallei* (www.ebi.ac.th).

### Recombinant proteins expression and purification

A successful transformant, *E. coli* BL21(DE3) containing pET24a-OmpABT, was grown overnight in a 5 ml LB broth containing 50 µg ml^−1^ kanamycin at 37 °C with shaking. The overnight culture was used to inoculate 200 ml fresh LB broth (50 µg ml^−1^ kanamycin) at 1:100 dilution and cultured at 37 °C to an OD_600nm_ of 0.6–0.8. Isopropyl-β-d-thiogalactoside (IPTG; Fermentas, Life Sciences) was added to the final concentration of 1 mM to induce protein expression. The induced culture was allowed to grow for 2 h at 37 °C with shaking at 200 rpm before the cells were harvested by centrifugation at 6000 rpm for 10 min at 4 °C. The cell pellet was resuspended in 5 ml of lysis buffer (IMAC5 pH 8.0) containing 20 mM Na_2_HPO_4_, 1 M NaCl, 10 % (v/v) glycerol, 5 mM imidazole and 15 µl of protease inhibitor cocktail (Roche applied Science) and lysed by sonication. After cell disruption, the resulting preparation was separated into soluble and insoluble or inclusion body fractions by centrifugation at 13,000 rpm for 30 min at 4 °C. The inclusion body was solubilized in IMAC5 containing 8 M urea and 5 mM dithiothreitol (DTT) overnight at room temperature under orbital agitation. Any insoluble materials were removed by centrifugation at 13,000 rpm for 60 min. The solubilized inclusion body was purified by IMAC under denaturing conditions using TALON® metal affinity resins (Clontech Laboratories Inc.). The equilibrated resins were incubated with the solubilized inclusion body for 3–4 h at room temperature under orbital agitation and then were loaded on a column. The column was continuously washed with 20 column volume (CV) of equilibration solution, 10 CV of IMAC10 (20 mM Na_2_HPO_4_, 1 M NaCl, 10 % (v/v) glycerol, 8 M urea and 10 mM imidazole), 10 CV of IMAC15 (20 mM Na_2_HPO_4_, 1 M NaCl, 10 % (v/v) glycerol, 8 M urea and 15 mM imidazole) and the final step with 10 CV of IMAC20 (20 mM Na_2_HPO_4_, 1 M NaCl, 10 % (v/v) glycerol, 8 M urea and 20 mM imidazole). The bound protein was eluted with 5 CV of 20 mM Na_2_HPO_4_, 1 M NaCl, 10 % (v/v) glycerol, 400 mM imidazole, 8 M urea, and 5 mM DTT. The elution fraction was concentrated using Amicon® Ultra centrifugal filter (Millpore Corporation). The protein concentration was determined by Bradford protein assay (Bio-Rad Laboratories Inc.). The truncated flagellin protein (FLAG300) was expressed and purified as describe in the previous report (Wajanarogana et al. 2013).

### SDS-PAGE and western blot analysis

The expressed and purified proteins were analyzed by SDS-PAGE according to the method described by Laemmli (1970) with 12 % polyacrylamide gel. After proteins were separated on the gels by electrophoresis, they were stained with Coomassie brilliant blue R-250 or electrophoretically transferred onto a polyvinylidene difluoride (PVDF) membrane (Pall Corporation) using Trans-blot®SD semi-dry transfer cell (Bio-Rad Laboratories Inc.). The blotted membrane was washed twice with TBS buffer (10 mM Tris–HCl pH 7.5, 150 mM NaCl) and blocked with blocking buffer (0.25 g blocking reagent in 50 ml of blocking reagent buffer, QIAexpress® detection assay) for 1 h at room temperature. After twice washing with TBST (20 mM Trish HCl pH 7.5, 500 mM NaCl, 0.05 % (v/v) Tween 20 and 0.2 % (v/v) Triton X-100), 1:1000 diluted anti-his HRP conjugate in blocking buffer was added and incubated for 1 h. Prior to protein band detection, the membrane was washed twice with TBST and one time with TBS buffer. Immunoreactive protein band was visualized with 3,3′,5,5′-tetramethylbenzidine (TMB) (KPL).

### Indirect ELISA and its evaluation

Prior to evaluate the potential of recombinant proteins as antigens for serodiagnosis of melioidosis with indirect ELISA, the optimal concentration of this assay reagents was determined by checkerboard titration method using pooled of five culture confirmed-positive of melioidosis sera and five sera of healthy donors. It was performed in duplicate using Microlon^®^ plates (Greiner bio-one). The 96-well microtiter plates were separately coated with 100 µl of 0.1 µg ml^−1^ of recombinant OmpABT purified protein antigen and 100 µl of 1 µg ml^−1^ of purified truncated flagellin fragment in coating buffer (carbonate/bicarbonate buffer, pH 9.6) at 4 °C for overnight. The plates were blocked with 150 µl of blocking buffer (0.5 % of bovine serum albumin in coating buffer) at 37 °C for 1 h and they were washed 3 times with PBS-Tween solution [PBS and 0.05 % (v/v) Tween 20)]. After washing, 100 µl of diluted serum samples (1:3200) in PBS were added in each well and then the plates were further incubated at room temperature for 1 h. The wells were washed with PBS-Tween solution 3 times and added with 100 µl of diluted (1:1000) anti-human IgG conjugated with HRP (KPL). After 1 h incubation at 37 °C, the wells were washed with PBS-Tween solution 3 times and 100 µl of TMB substrate solutions were added to each well. The reaction was left to develop for 15 min and stopped with 100 µl of 1 N HCl. The reaction products were determined using a microplate reader at OD_450nm_ (Bio-Rad model 550; Bio-Rad Laboratories Inc.). On each plate, combinations of positive (pooled positive) and negative controls (direct conjugate control) were carried out in duplicate.

### Data analysis

The mean and standard deviation of normal serum samples were calculated and cut-off value was determined as the mean of normal controls plus two standard deviations (SD). Serum samples were considered positive if the mean optical densities were higher than cut-off point. For consideration as positive following the criteria of multiple-antigen ELISA, the serum tested specifically react with any antigen when the cut-off value was calculated from the mean optical densities plus three standard deviations (Zhang et al. 2009). Statistical analysis was performed with GraphPad Prism version 5.0.

## Results

### *Omp*A gene cloning and DNA sequencing

**Fig. 1:**
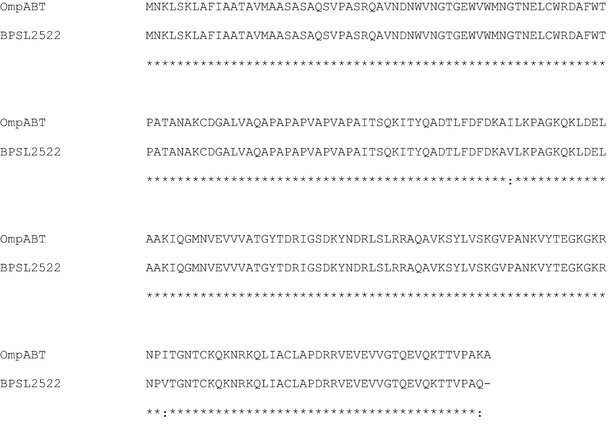
A comparison of deduced amino acid of OmpABT and the published OmpA protein of *B. pseudomallei*, BPSL 2522, *asterisks* represent identity and *dots* indicate similarity

The amplified product, 680 bps, was isolated from genomic DNA of *B. thailandensis* E264 by specific primers designed based on the *ompA* sequence of *B. pseudomallei* K96243. The purified amplicon was successively digested with *Nde*I and *Eco*RI and cloned into *Nde*I/*Eco*RI linearized pET24a(+) to construct the recombinant plasmid (pET24a-OmpABT). After transformation into *E. coli* BL21(DE3), a clone was selected to sequence and analyze. The nucleotide sequence showed a high degree of identity (98–100 %) to the OmpA family protein of *B. thailandensis* and *B. pseudomallei* when examined by blastn (data not shown). A comparison of deduced amino acids sequence with the published OmpA protein of *B. pseudomallei* (BPSL 2522) revealed that they have about 99 % homology over the entire sequences (Fig. 1).

### Analysis of expressed recombinant protein

**Fig. 2:**
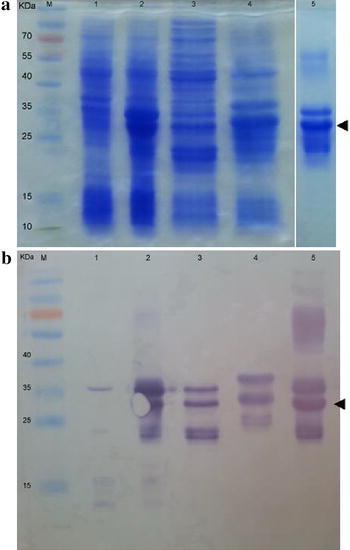
The expressed and purified OmpABT was confirmed by 12 % SDS-PAGE (**a**) and western blotting (**b**). *Lanes 1* and *2*, total cell lysate of *E. coli* harboring pET24a-OmpABT of uninduced and induced clone with IPTG, respectively, *Lane 3*, soluble fraction, *Lane 4*, inclusion bodies fraction, *Lane 5*, concentrated eluted protein and Lane M, protein molecular weight marker. Relative molecular mass (kDa) of standard proteins is shown on the left-hand side. The *arrowhead* indicates the location of targeted protein

The selected clone carrying the construct pET24a-OmpABT plasmid was induced with 1 mM IPTG for 2 h at 37 °C. The expressed proteins of about 27 and 35 kDa appeared in the total cellular proteins as demonstrated by SDS-PAGE analysis; however, they mainly resided within inclusion bodies (Fig. 2a). The identity of the expressed protein was confirmed by Western blot analysis using anti-his antibody (Fig. 2b). The lower immune-reactive bands (less than 25 kDa) were observed and a less abundant higher molecular weight band (about 60 kDa) suggests that they present an internal degradation and a dimer of recombinant proteins (Allwood et al. 2008; Hara et al. 2009), respectively. Prior to protein purification under denaturing condition, the inclusion bodies was initially solubilized with 8 M urea and reduced with DTT and then insoluble material was removed by centrifugation. The soluble fraction was purified by Talon™ resins directed to the hexa-histidine tag at the carboxyl terminus of the recombinant protein based on immobilized metal affinity chromatography (IMAC). Step-wise washing not only helped to get rid of non specific proteins but the targeted proteins were also lost (data not shown). The bound proteins were eluted with elution buffer and concentrated for use in the further step (Fig. 2). Purified recombinant protein was found to be 3.65 mg from 200 ml of culture or 18.25 mg l^−1^.

### Evaluation of constructed OmpABT and FLAG300 proteins as serodiagnostic reagents using indirect ELISA

**Fig. 3:**
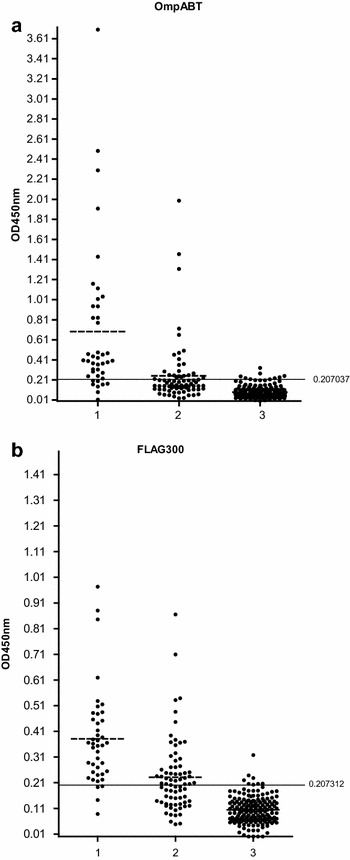
The reactivity of serum samples with OmpABT (**a**) and FLAG300 antigens (**b**) in indirect ELISA. Both antigens were screened with culture confirmed-melioidosis sera (1), disease control sera (2) and healthy donor sera (3). *Each symbol* represents the average of duplicate readings of one serum samples. Cut-off values were showed by solid lines and mean of each group (*dash lines*)

Both recombinant antigens were used in this assay to detect antigen-specific IgG antibody in three groups of sera: bacterial culture confirmed-melioidosis sera, septicemia with other bacteria serum samples (diseases control) and sera of blood donors from endemic areas (Amnat Charoen and Khon Khaen provinces) and non-endemic region (Pathumthani province). The optimal cut-off point of individual recombinant antigens (mean plus two standard deviations) were calculated from absorbance values of serum samples from healthy donors (n = 167). They were found out to be 0.207037 and 0.207312 for OmpABT and FLAG300, respectively. The average of optical densities (ODs) of endemic and non-endemic serum samples are not significantly different. The OD values observed with of all the 3 sera groups and cut-off values are given in Fig. 3. The OD value of a serum sample greater than the cut-off point was considered as positive. The serodiagnostic indices of indirect ELISA using individual antigens based on defining the culture confirmed-positive sera and healthy donor and disease control sera as true positive and true negative, respectively were calculated (Table 1). The sensitivity of FLAG300 *B. thailandensis* was higher (90.48 %) than OmpABT (80.95 %) recombinant protein whereas the specificity was very similar (87.14 and 89.21 %) as well as the accuracy (around 88 %). Using a combination of two recombinant proteins to predict the potential for serodiagnosis of melioidosis, based on analysis of the individual reactivities of FLAG300 and OmpABT with the serum samples, was 85.71 % sensitivity and good specificity (92.95 %).

**Table 1:** Sensitivity, specificity and accuracy of indirect ELISA using OmpABT and FLAG300 antigens

Diagnostic index	Serodiagnostic reagent		
	OmpABT	FLAG300	OmpABT and FLAG300 combination
Sensitivity^a^	80.95 (34/42)	90.48 (38/42)	85.71 (36/42)
Specificity^b^	89.21 (215/241)	87.14 (210/241)	92.95 (224/241)
Accuracy^c^	87.99 (249/283)	87.63 (248/283)	91.87 (260/283)

^a^(No. of positives of melioidosis sera/total no. melioidosis sera) × 100

^b^(No. of negative of non-melioidosis sera/total no. non-melioidosis sera) × 100

^c^(No. of positives of melioidosis sera and negatives of non-melioidosis sera/no. of all tested sera) × 100

## Discussion

The diagnosis of melioidosis can be done within 3 strategies, bacteriological, molecular and serological methods. Even though bacterial culture is the reference method so far, it has many limitations such as it is time consuming, requires good practice and a high level of experience of the investigator, and it is a relatively low sensitivity assay (Limmathurotsakul et al. 2010). Septicemic melioidosis patients might expire before a laboratory report, and thus early detection is imperative. A number of serological tests being developed to improve diagnosis of melioidosis using both recombinant proteins and extracted antigens from *B. pseudomallei* (Allwood et al. 2008; Anandan et al. 2010; Parthasarathy et al. 2008). In previous reports, the principal immunogenic proteins of *B. pseudomallei* are located on the cell surface such as OmpA or Omp3, truncated flagellin proteins and type IV pilus protein (PilO) (Allwood et al. 2008; Chen et al. 2003; Essex-Lopresti et al. 2005). However, such reports reveal quite high diagnostic indices, and serious biosafety level containment facilities (BSL-3) are necessitated to develop such assays. Our previous report demonstrated that the flagellin protein fragment of the closely related species, *B. thailandensis*, can react with antibodies in positive serodiagnostic sera of melioidosis (Wajanarogana et al. 2013). Moreover, non-pathogenic *B. thailandensis* was used as a potential model to refer certain biological properties of *B. pseudomallei* because they are highly similar at the genetic level (Yu et al. 2006). The experimental manipulation of non-pathogenic strain can be performed under standard routine microbiological condition (BSL-1). In the present study, we produced recombinant proteins, OmpABT and FLAG300 in order to evaluate their potential as antigens for diagnosis of melioidosis by an indirect ELISA. The recombinant proteins were overexpressed as incorrectly folded insoluble products (Lilie et al. 1998). The expressed OmpABT does not require a refolding process, however, since there is recognition of linear epitopes. The recombinant proteins fused to hexa-histidine tag at the carboxyl termini were single step purified under denaturing conditions with metal affinity resin chromatography. Although stringent washing conditions were carried out, multiple protein bands were detected. However, those bands strongly react with anti-hexa-histidine antibody indicating that they correspond to the fusion protein. The distinct bands with higher and slightly lower molecular weight than targeted recombinant protein might be the results of different isoforms and proteolytic breakdown, respectively (Allwood et al. 2008; Hara et al. 2009).

Several previous studies demonstrate that truncated flagellin fragment and OmpA protein of *B. pseudomallei* serve as the diagnostic potential of melioidosis (Allwood et al. 2008; Arora et al. 2015; Chen et al. 2003; Hara et al. 2013). In this study, the recombinant proteins, OmpABT and FLAG300, from the closely related species, *B. thailandensis*, were evaluated as antigens which specifically react to melioidosis antibodies using indirect ELISA and we calculated the serodiagnostic indices. The evaluations were performed with 42 culture confirmed-melioidosis sera, 74 other diseases control sera and 167 healthy donor sera. It is not surprising that the average absorbance of the two recombinant proteins was significantly higher in melioidosis patients sera compared to non-melioidosis sera (data not shown). The sensitivity (90.48 %) of ELISA with FLAG300 was found to be higher compared to a previous report (82.7 %) whilst the specificity (87.14 %) was slightly lower (94.6 %) (Wajanarogana et al. 2013). The previous study used clinically suspected melioidosis sera with serological positive as a reference samples but in this work we used bacterial culture-confirmed sera. Moreover, the numbers of normal serum samples were quite different (40 and 167 samples). An ELISA using the truncated flagellin antigen has been considered in non endemic area by Chen et al. (2003), which demonstrated high sensitivity (93.8 %) and specificity (96.3 %).

Various studies revealed a sensitivity of ELISA with recombinant OmpA antigen from *B. pseudomallei* between 59–95 and 90–98 % specificity, which were considered at various cut-off points (Allwood et al. 2008; Arora et al. 2015; Hara et al. 2013). In this study, firstly, we detail the use of recombinant OmpA of *B. thailandensis* (OmpABT) as the potential antigen for diagnostic of melioidosis by developing indirect ELISA. At the cut-off value (mean plus 2SD), good sensitivity (80.95 %) and specificity (89.21 %) were gained which substantially improved over the clinical standard indirect hemagglutination test (IHA). Because the carboxyl-termini of OmpAs are quite conserved within enteric bacteria and *B. pseudomallei* and contain the immunodominant epitope that is recognized by antibodies formed during the course of infections (Hara et al. 2009; Jeannin et al. 2002; Puohiniemi et al. 1990), infection with these bacteria raises antibody which cross react with OmpABT protein causing reduced specificity. The variable region of recombinant protein could be utilized to increase the specificity of the test although sensitivity might be reduced.

A novel approach using more than a single antigen to improve serodiagnosis of melioidosis has revealed that sensitivity is improved whilst retaining good specificity (Hara et al. 2013). In this study we demonstrate better sensitivity (85.71 %) than OmpABT alone and improved specificity compared to either alone (92.95 %). Besides their utility in the diagnosis of melioidosis, these recombinant antigens should also be considered in the design of a vaccine against *B. pseudomallei* infection as they induce an immune response (Allwood et al. 2008; Hara et al. 2009; Ye and Gan 2007).

## Conclusion

Here, we suggest that the potential antigens of non-pathogenic *B. thailandensis* could be utilized to develop serodiagnosis of melioidosis within a single protein antigen or double antigens ELISA to eliminate health risks to laboratory personnel for Tier-1 agent exposure.
